# Development and comparative analysis of machine learning algorithms for predictive atmospheric corrosion modeling

**DOI:** 10.12688/openreseurope.19770.1

**Published:** 2025-03-27

**Authors:** Jose Manuel Perales Fernández, María López Abelairas, Arturo Sánchez-Ramos, Lila Otero-Gonzalez

**Affiliations:** 1 Idener Research and Development A.I.E., La Rinconada, Sevilla, 41300, Spain

**Keywords:** Atmospheric corrosion prediction, grid search, hyperparameter tuning, corrosion dataset, machine learning algorithms

## Abstract

**Background:**

Industrial content and infrastructure are in constant danger from atmospheric corrosion, which affects economies globally. However, there is a lack of a consistent set of comprehensive data that completely surrounds the range of this problem in diverse climate and locations. The purpose of the research is to evaluate the factors that contribute to atmospheric corrosion and its diverse effects on materials in various environments.

**Methods:**

By creating a comprehensive dataset by collecting and standardizing corrosion data from diverse environments and geographic regions and initially analyzing the data, it helped indicate the main parameters affecting corrosion. This guided the selection of future features for further modeling. Several machine learning algorithms were tested, such as linear regression, decisions tree, neural network, and, most especially, attempting methods, for their corrosion rate prediction capabilities. These models were assessed based on their prediction’s accuracy, and computational efficiency, with special attention to refining their performance through detailed feature engineering and hyperparameter adjustment.

**Results:**

Upon evaluating the performance of conventional predictive models, the research indicated that the machine learning approaches, especially with random forests methods of dress, were excellent in predicting corrosion rates, significantly improved upon these capabilities. By analyzing various machine learning approaches, it became clear that it was important to enhance their accuracy by selecting the best features and customizing them.

**Conclusions:**

This work represents a significant advancement in the predictive modeling of atmospheric corrosion. It highlights the invaluable role of machine learning in this field. By integrating varied data sets and applying sophisticated machine learning techniques, it has established a foundation for ongoing research and the practical application of corrosion management strategies. The exceptional performance of ensemble methods, like random forests, signals their potential to improve prediction capabilities and guide more effective corrosion prevention measures.

## Plain Language Summary

This paper addresses the issue of atmospheric corrosion, a naturally occurring phenomena that gradually deteriorates materials exposed to air over time, jeopardising industrial facilities, bridges, and buildings. One of the most significant obstacles in capturing and forecasting this type of corrosion is a lack of structured data. We collected and standardised corrosion data from several locations and environmental scenarios to create a more comprehensive and meaningful dataset to help with this.

This knowledge enabled us to study which elements contribute the most to corrosion and influence our selection of relevant data for constructing prediction models. Following that, we explored with a variety of machine learning algorithms (computer-based methods for detecting patterns in data), ranging from simple linear regression to more complicated decision trees and neural networks. Following a comparison of their accuracy and efficiency, we noticed that some approaches, particularly those that contain multiple models known as "ensemble methods," such as random forests, performed best in predicting how quickly materials would corrode.

We also found that improving these models by carefully refining the proper data inputs and changing the settings of the model allows them to provide more accurate predictions. This study not only reveals which machine learning algorithms are best suited for corrosion prediction, but it also lays the framework for better monitoring and protection of materials exposed to the atmosphere. This work promotes the overall objective of minimising material degradation and ensuring the longevity of critical infrastructure by combining data from numerous sources and employing a variety of analytical techniques.

## Nomenclature

A                Parameter obtained from least-squares method for Klinesmith’s model

B                Parameter obtained from least-squares method for Klinesmith’s model

b _i_                Regression coefficients

C                TOW mean environmental parameter in Klinesmith’s model

Cl ^-^              Chloride concentration

CR             Corrosion depth

D                Parameter obtained from least-squares method for Klinesmith’s model

D                Corrosion depth in µm for ISO 9224 model

E                 SO _2_ environmental parameter in Klinesmith’s model

e                  Euler's number (value: 2.71828)

F                  Parameter obtained from least-squares method for Klinesmith’s model

G                 Cl ^-^ environmental parameter in Klinesmith’s model

h                  Metal-environment-specific time exponent in ISO 9224 model

H                 Parameter obtained from least-squares method for Klinesmith’s model

J                  Parameter obtained from least-squares method for Klinesmith’s model

n                  Number of observations

R ^2^                Coefficient of Determination

R _corr_            Corrosion rate in ISO 9224 model

RMS            Root Mean Square Error

SO _2_              Sulphur dioxide concentration

t                    Exposure time

T                   Temperature

T _0_                 Mean temperature in Klinesmith model

TOW            Time of wetness

y                    Dependent variable (corrosion depth)

$\hat{y_{\text{ι}}}$ Predicted value for the i-th observation

$\bar{y}$ Mean of the actual values

y _i_                   Actual value for the i-th observation

## 1 Introduction

Steel and other metallic alloys provide excellent mechanical qualities while remaining reasonably affordable. However, they have a significant weakness: they are susceptible to corrosion. This phenomenon, defined as the progressive deterioration of materials produced by chemical, electrochemical, or other reactions with their environment, is a critical issue in a wide range of industries, including manufacturing, infrastructure, and transportation ^1^. Its economic impact is enormous, requiring costly repairs, maintenance, and replacements. Furthermore, corrosion can compromise the structural integrity and safety of infrastructures, posing significant risks. Understanding and preventing corrosion is, therefore, critical to assuring the durability and reliance of materials in a wide variety of applications.

Depending on the environmental conditions and the material qualities, several mechanisms of corrosion may occur, namely electrochemical corrosion ^2^, galvanic corrosion ^3^, microbiologically influenced corrosion (MIC) ^4^ and passivation ^5^, demonstrating the complexity of the effect. To deeply understand these phenomena, modelling has resulted in a pivotal approach to studying and predicting corrosion behavior under different circumstances. These techniques have experienced notable advancements over the decades, bringing unique insights and tools to the field.

Initially, analytical models such as Finite Element Analysis (FEA) and Computational Fluid Dynamics (CFD) played a central role. In the case of FEA, a computational method solves equations governing ion movement and electrochemical reactions, providing detailed spatial and temporal insights into corrosion processes. Studies such as the one by Izquierdo *et al.* ^6^ have shown how FEA can mimic the intricate details of corrosion progression, offering a profound understanding of material degradation over time. Similarly, CFD models have been invaluable in examining how corrosive agents like oxygen and ions travel within fluids, influence corrosion rates and distribution. These models help to predict the impact of fluid dynamics on corrosion in environments like pipelines ^7^ and offshore structures and compute corrosion rates and distribution. Another mechanistic approach, the phase field method, is used to simulate phase transitions and microstructural evolution in materials, providing a microstructural perspective on corrosion processes ^8^. Additionally, atomic-scale modelling, including molecular dynamics and density functional theory (DFT), offers insights into the fundamental atomic-scale processes that drive corrosion, such as adsorption, electron transfer, and surface reactions ^9^.

However, mechanistic and first principles models have certain limitations that make them less practical for large-scale predictive applications. These models are highly computationally demanding, often requiring significant processing power and time to generate results. Additionally, they are not inherently predictive, meaning they are limited in their ability to forecast corrosion behavior in new scenarios without prior detailed knowledge of the specific corrosion process and material properties. Furthermore, their focus is often on microscopic scales, which may not translate well to insights applicable to large, real-world infrastructures. These models also struggle to handle large amounts of complex data, making them less suited for big data applications. Consequently, these drawbacks highlight the potential for machine learning models, which can address these challenges by offering scalable, data-driven solutions that are capable of predicting corrosion outcomes without the same level of computational or domain-specific knowledge.

Probabilistic models are strong tools to address these uncertainties inherent to corrosion processes. These models consider variability in material properties, environmental conditions, and other factors, providing a probabilistic assessment of corrosion risk and remaining service life ^10^. The Monte Carlo simulation is a prominent example of this approach. By incorporating a range of environmental variations and material diversities, Monte Carlo simulations offer a comprehensive view of corrosion behavior, as in the model proposed by Engelhardt *et al.* ^11^. These probabilistic models have been crucial in industries where understanding and mitigating the risk of corrosion is essential for safety and economic efficiency. The main disadvantages are the computational demanding time required for more complex models ^12^.

In recent years, the probabilistic approach has evolved into data-driven and machine learning (ML) models, introducing a new promising approach to corrosion modelling. Leveraging extensive datasets, ML models can recognize intricate patterns related to corrosion, make accurate predictions about corrosion rates, and fine-tune strategies for prevention and mitigation. Models such as the one from De Masi *et al.* ^13^ have demonstrated the significant potential of ML in this field. This approach not only enhances the accuracy of corrosion predictions compared to previous models but also allows for real-time monitoring and adaptive maintenance strategies, which make them very suitable to forecast and enable effective corrosion control measures.

Following this line, the present work studies a set of ML algorithms for understanding and predicting corrosion processes for offshore conditions based on several environmental parameters. This work focuses specifically on atmospheric corrosion as one of the main problems that must be understood, in order to reduce the economic impact of replacing damaged materials and extend the lifespan of structures. To achieve this, the corrosion depth suffered by steel in atmospheric conditions will be modelled based on environmental parameters ^14^ such as temperature (T), relative humidity (RH), time of wetness (TOW), sulphur dioxide deposition rate (SO _2_), chloride deposition rate (Cl ^-^), and exposure time. The techniques used represent a broad range of approaches of various levels of complexity, all related to ML, to anticipate corrosion occurrences precisely under a wide variety of climate circumstances and variables. The findings of this study are offered to help design more effective corrosion prevention and mitigation strategies.

## 2 Methods

For the development of the ML algorithms, a work methodology divided into 7 steps was established (Figure S1 from Extended data ^15^). The first step focused on the construction of the dataset ( **Data collection**). This involved searching the literature to collect the largest amount of data related to environmental corrosion and constructing one unified dataset. The second part focused on data preprocessing ( **Data preprocessing**). Then, the dataset was separated into different sub-datasets: one for training, another for validation and a final one for testing ( **Data splitting**). The fourth step consisted of the development of the algorithm architecture by testing different ML algorithms ( **Model development**). Additionally, conventional models were developed in parallel for later comparison ( **Conventional models**). For testing the architecture of the different ML parameters, calibration was done following accuracy metrics and rebuilding the model with new model parameters ( **Calibration**). Sixth, the results obtained were analysed, looking for the model with the best predictive capacity ( **Analysis of results**). Finally, the model was implemented and tested, comparing its predictive capacity with conventional models ( **Model implementation**).

### 2.1 Data collection

***2.1.1 Literature search***

Data was collected from various publications, including scouring academic journals, research papers, industry reports, and other publications related to corrosion. These publications cover a broad spectrum of metals and environmental conditions, making the dataset diverse and valuable. In this study, carbon steel samples were selected.

The first step was to define the parameters involved in each publication to have an overview of what kind of data can be expected, where T in degrees, RH in %, TOW in hours per year, SO _2_ in mg/m ^2^·d, Cl ^-^ in mg/m ^2^·d, rain precipitation (P) in mm of water and time in years. Data of corrosion measurements were usually in µm or µm/year, depending on whether the data represented a time series or just a single measurement after 1 year.

After that, rows with formal defects or duplicates were located and removed. As the corrosion study is centred on offshore conditions, categories C5 and CX of corrosion were inspected following ISO 12944-2 guidelines ^16^. All the information collected for the dataset construction and the parameters involved are detailed in Table S1 from Extended data ^15^.

***2.1.2 Construction of the dataset***

For the construction of the final dataset, several datasets were first constructed considering different configurations of input parameters (T, RH, TOW, SO _2_, Cl ^-^, P and time), the total amount of data and the percentage of C5-CX corrosion category data of each dataset, removing the duplicates of them, showing the final configuration on Table 1. Most of the corrosion data collected refers only to the first year of corrosion, but some publications reported time series up to 12 years that were also included considering the year as an additional input. After evaluating the constructed datasets, it was found that the percentage of C5-CX corrosion data was similar in all datasets, and therefore, the dataset with the larger size (Dataset 4) was selected for the subsequent model development. Based on this dataset, T, TOW, SO _2_ deposition rate, Cl ^-^ deposition rate and time of exposure were considered as input parameters, while in years, the corrosion depth (CR in µm) was considered as the output parameter.

**Table 1.** Overall description of the different built datasets, indicating the input parameters, being that ones T, RH, TOW, SO _2_, Cl ^-^, P and time (marked with X) that contain each dataset; the total dataset size (number of rows) and the number and percentage of data corresponding to the corrosive categories C5-CX according to ISO 12944-2 guidelines.

	T	RH	TOW	SO _2_	Cl ^-^	P	time	Total size	C5-CX	% C5-CX
Dataset 1	X	X	X	X	X	X		198	27	13.64
Dataset 2	X	X	X	X	X			243	32	13.17
Dataset 3	X		X	X	X			595	67	11.26
Dataset 4	X		X	X	X		X	816	99	12.13
Dataset 5			X	X	X			662	72	10.88

This dataset of 816 records is composed of 180 records from the MICAT project (130 record obtained from Pintos *et al.* ^14^, 46 records from Chico *et al.* ^17^ and 4 records from Panchenko *et al.* ^18^), 190 records from the ISOCORRAG project (Chico *et al.* ^17^), 395 records obtained from Cai *et al.* ^19^, 6 records from Castaño *et al.* ^20^, 7 records from Hou *et al.* ^21^, 25 records from the E-Asia projects ( *Table IV-3* from To *et al.* ^22^) and 13 records from EFC ^23^ (the data from 24, 25 and 26 in Table S1 from Extended data ^15^ finally were not included in the final dataset). 

### 2.2 Preprocessing

***2.2.1 Data cleaning and filtering process***

A comprehensive data preprocessing involving data cleaning and filtering steps was put in place. The primary target was to ensure the integrity and reliability of the dataset for subsequent analysis. As a critical phase of this process, a statistical analysis aimed at detecting the presence of outliers within the dataset was conducted. To achieve this, the Isolation Forest ^27^ algorithm was used as the first step.

After the data preprocessing phase (cleaning), the statistical analysis consisted of an examination of various statistical parameters, including mean, standard deviation (std), and percentiles. To further explore the relationships among the model parameters, a Pearson's correlation matrix was constructed (Figure S2 from Extended data ^15^).

### 2.3 Partition of the dataset in training, validation and testing sub-datasets

***2.3.1 Technique employed on the dataset partition***

Dividing the dataset into training, validation, and testing sub-dataset is essential for developing robust, reliable, and generalizable ML algorithms. It supports model development, hyperparameter tuning, prevents overfitting, and allows for unbiased performance assessment. The train_test_split ^28^ function of the Python sklearn library was used to randomly divide the full dataset into the subsequent 2 sub-dataset. To verify that the data distribution was adequate, the algorithm was executed 100 times. In each iteration, it was verified that the subsets assigned reflected the same distribution of the time exposure years for each sub-dataset. This verification showed that each time, for each range of time exposure (1 year, 2 years, etc.), same amount of rows (80% and 20% for each time exposure) were distributed between both sub-datasets, with different row values each time (splitting randomly). The train_test_split function was applied to split the overall dataset into one sub-dataset containing the test data ( *test dataset*) and another sub-dataset containing the training and validation data together ( *training+validation dataset*), as during the model development, the training and validation dataset was divided randomly several times in training dataset and validation datasets. The *test dataset* was left untouched to test the developed models once optimized.

### 2.4 Model’s development and their architectures

The development of the ML model was focused on an analysis of 6 different types of algorithms to evaluate which one can achieve a better fitting and predictive capacity. The application of these algorithms was in increasing order of complexity, starting from the simplest one and progressing to the most complex one. The objective of developing different algorithms and not just focusing on one was to avoid any assumption that could result in the development of an algorithm that was not the best one according to the means available.

***2.4.1 Methods employed for definition of the architecture of each model***

**Multiple Linear Regression**

The first algorithm analysed was multiple linear regression (MLR) ^29^. MLR is a statistical technique used to analyse the relationship between a dependent variable, in this case, CR, and two or more independent variables such as T, TOW, RH, SO _2_, Cl ^-^ and exposure time. The goal of this analysis was to understand the relationship between the dependent variable and the independent variables and to use this relationship for predicting the CR based on the environmental features.

For modelling the MLR the data must be first normalized. We chose a Min-Max normalization. Min-max normalization is a data preprocessing technique used to scale the data values in a range of 0 to 1. To apply min-max normalization to the variables, the minimum and maximum values of each variable were found and then the following formula was applied:


Scalevalue=(originalvalue−min⁡value)/(max⁡value−min⁡value).(1)

The next step was to build a regression model by fitting the selected independent variables to the dependent variable. The equation for the regression model is the following one:


$$y=b_{0}+b_{1}T+b_{2}TOW+b_{3}SO_{2}+b_{4}Cl^{-}+b_{5}t,\;(2)$$

where *y* is the dependent variable (CR); *b _0_* is the intercept; *b _1_*, *b _2_*, *b _3_*, *b _4_* and *b _5_* are the regression coefficients for each independent variable (environmental features); *T*, *TOW*, *SO2*, *Cl ^-^* and *t* are the normalized independent variables (environmental features). To remove the variance involved in building the model by using a random partition of the training data set and the validation set, the model was analysed 100 times, obtaining the average of the accuracy parameters for later comparison with the other models.

**Polynomial Regression**

The subsequent algorithm under investigation was polynomial regression (PR) ^30^, a widely employed technique in ML designed to establish nonlinear relationships between dependent and independent variables. In this context, it focused on crafting a polynomial linear regression model tailored to predict CR. The same normalization method applied in the MLR was used. A critical decision entailed the determination of the polynomial algorithm's degree. This investigation was initiated by fitting a polynomial model of degree 2 and systematically increasing the degree to 4 to achieve an optimal fit for our dataset. For each progressive polynomial degree, the dataset was partitioned into distinct training and validation sets. The training set served for model training, while the validation set assessed the model's performance. Employing the same criteria as in the MLR context to mitigate dataset split variance, 100 distinct analyses were executed across a range of polynomial degrees.

**Decision Tree Regressor**

The third algorithm under investigation was the decision tree regressor (DTR) ^31^. DTR is a hierarchical model that employs a tree-like structure to make decisions regarding specific outcomes. Each node within the tree corresponds to a decision based on a particular feature or variable. As the tree expands and branches, each decision becomes increasingly specific, ultimately culminating in a prediction concerning the outcome.

To initiate the development of a DTR model, the first step entailed dividing the data into a training set and a validation set. In this case, data normalization was not applied as the prediction is discrete despite the continuous output. The training set serves as the foundation for model construction, while the validation set assesses the model's accuracy. To further enhance the model's robustness and generalizability to new data, a cross-validation ^32^ technique was implemented, dividing the dataset into five parts.

Subsequently, the determination of hyperparameters for the DTR model was carried out, with a primary focus on two key hyperparameters: i) the maximum depth of the tree and ii) the minimum number of samples required to split a node ( Table 2). The maximum depth of the tree dictates how deeply the tree can extend before ceasing to branch further. Conversely, the minimum number of samples necessary to split a node specifies the threshold for the minimum number of samples required within a node before it can be split. The grid search algorithm was executed 100 times to identify optimal hyperparameter values within a defined value range ( Table 2).

**Table 2.** DTR hyperparameters studied.

Hyperparameter	Value range values
Maximum depth	1–10
Minimum number of samples	10–60 (step = 10)

**Random Forest Regressor**

The fourth algorithm analysed was random forest regressor (RFR) ^33^. In RFR, several decision trees are built, and their outputs are combined to obtain the final prediction. The number of trees used in the random forest is determined by the hyperparameter number of estimators. Three hyperparameters were tuned in the RFR model ( Table 3): i) maximum depth tree, ii) number of estimators, and iii) minimum number of samples required to split an internal node. The range of values studied are listed in Table 3. The maximum depth tree controls the depth of the individual decision trees being built, which helps to reduce overfitting. The number of estimators hyperparameter controls the number of decision trees to be built, which can improve the model's performance. The minimum number of samples required to split internal nodes helps to prevent further splitting in some of the nodes and aids in reducing the model's computational burden. A cross-validation technique was used for finding the best hyperparameters composition. The grid search algorithm was run 100 times with training and validation datasets, split them from the main dataset randomly each time, applying a cross-validation five times per iteration to minimize the influence of training data on the model architecture.

**Table 3.** RFR hyperparameters studied.

Hyperparameter	Range values studied
Maximum depth	1–20
Minimum number of samples	5–60
Number of estimators	20–120

**Support Vector Regressor**

The fifth algorithm analyzed was support vector regressor (SVR) ^34^. The SVR is a type of supervised ML model used in regression tasks. It is used to predict continuous output variables using input features. The goal of SVR is to find a regression function that minimizes the error between predicted values and real ones with a margin of error (epsilon).

The development of an SVR involves selecting the right kernel, C factor, gamma, and epsilon ( Table 4). The kernel function transforms the input data into a higher-dimensional space, where it can be separated into different classes. The C factor is a regularization parameter that controls the trade-off between model complexity and training error. It helps in determining the amount of error allowed in the training process. A smaller C value indicates a softer margin, allowing more training data to be misclassified, while a larger C value produces a harder margin, requiring all training data to be correctly classified. Gamma is a parameter that defines the shape of the decision boundary. It controls the degree of influence of a training example on the decision boundary. A smaller gamma value results in a decision boundary with a higher curvature, whereas a larger gamma value results in a decision boundary with a lower curvature. The epsilon parameter defines the insensitive zone around the regression line, within which the model will not consider the errors. The selection of the epsilon value depends on the tolerance level for the error.

**Table 4.** SVR parameters studied.

Hyperparameter	Values studied
Kernel	Radial basis function, sigmoid
C	1–10
Gamma	0.01–10
Epsilon	0.01–0.5

The same normalization method as for MLR and PR algorithms was applied to the data. To optimize the model performance, the hyperparameters were tuned using cross-validation techniques. In this case, the computational analysis time was too high for repeating the grid search algorithm, so it was applied 5 times to find the best hyperparameter combination. The grid search algorithm parameters are listed in Table 4.

**Multi-Layer Perceptron Regressor**

The sixth and last algorithm analyzed was the multi-layer perceptron regressor (MLPR) ^14^. The MLPR neural network is a type of feedforward artificial neural network (ANN) that consists of multiple layers of perceptron (also known as nodes or artificial neurons) arranged in a series of interconnected layers. Each perceptron receives input signals, processes the information, and produces output signals that become inputs to the next layer's perceptron until the final output is produced.

The data was normalized employing the Min-Max normalization. Next, the design of the neural network architecture was carried out through a grid search algorithm applying cross-validation. The tuned hyperparameters were activation function, learning rate, alpha, solver and the number of hidden layers ( Table 5). The activation function introduces non-linearity into the model. The learning rate determines how fast the model learns from the training data. The alpha value controls overfitting though L2 regularization ^35^. The solver parameter is used to specify the optimization algorithm used in the mode. Finally, the number of hidden layers determines the complexity and capacity of the neural network regressor. For this case, the computational analysis time was the highest, so the grid search algorithm was run just one time. The values studied from the search algorithm are listed in Table 5.

**Table 5.** MLPR parameters studied (Lbfgs: Limited-memory Broyden–Fletcher–Goldfarb–Shanno; Sgd: Stochastic gradient descent).

Hyperparameter	Values studied
Activation function	identity, logistic, tanh, Rectified linear unit
Learning rate	constant, adaptive, invscaling
Alpha	0.001–0.11
Solver	adam, lbfgs, sgd
Hidden layers	(10,10,10) -(20,80,30) step layer (2,10,2)

***2.1.2 Description of the metrics employed for tunning each modelling hyperparameter***

The different combinations of hyperparameters of each algorithm were evaluated using the statistical indicators of root mean square error (RMSE) and R ^2^. Those hyperparameter configurations with the lowest RMSE and highest R ^2^ were the chosen configurations for each ML algorithm evaluated. R ^2^ ( Equation 3) is a statistical measure that quantifies the proportion of the variance in the dependent variable (target) that can be explained by the independent variables used in the model. It offers a simple and interpretable metric to gauge how well the model captures the variability of the target.


$$R^{2}=1-\frac{\Sigma {\left(y_{i}-{\hat{y}}_{i}\right)}^{2}}{\Sigma {\left(y_{i}-\bar{y}\right)}^{2}}\;(3)$$

RMSE ( Equation 4) represents the dispersion or spread of prediction errors, which are essentially the deviations of data points from the regression line. In essence, RMSE provides insights into the degree of data clustering around the optimal fit line, indicating how closely the data conforms to this line.


$$RMSE=\sqrt{\sum_{i=1}^{n}\frac{{\left({\hat{y}}_{i}-y_{i}\right)}^{2}}{n}}\;(4)$$

Here *y*_i_ is the real output value, $\hat{y_{\text{i}}}$ is the predicted output value, $\bar{y}$ is the mean output value and *n* is the total of rows.

## 3 Results and discussion

### 3.1 Data preprocessing

The results of the outlier detection process were compared with previous research findings (Table S1 from Extended data ^15^) that had identified and examined outliers within each specific dataset. This comparative analysis was carried out to determine whether it was appropriate to retain or exclude these identified outliers. After conducting a thorough examination and carefully comparing each row with its corresponding publication context, it was concluded to retain these outliers within the dataset since the previous publications had already identified and resolved any inconsistencies in the data. Consequently, although the Isolation Forest algorithm classified 121 data points as statistical outliers, these anomalies seemed to be artifacts stemming from the inherent complexities in the data derived from various sources and datasets.

The descriptive statistics ( Table 6) revealed an interesting observation concerning the CR parameter, which represents CR in µm. At first glance, the maximum CR value (1,804.4 µm) seemed to deviate significantly from conventional statistical indicators (Q3 = 69.7 µm), suggesting it might be an outlier. However, the examination using the Isolation Forest algorithm definitively determined that this value did not meet the criteria for an outlier. This finding held true for other dataset parameters, except for temperature, where all statistical indicators consistently aligned.

**Table 6.** Summary of descriptive statistics of the full dataset ( *dataset 4)* used for model development.

Index	Temperature (ºC)	TOW (h/year)	SO _2_ (mg/m ^2^·d)	Cl ^-^ (mg/m ^2^·d)	time (years)	CR (µm)
count	816.00	816.00	816.00	816.00	816.00	816.00
mean	13.93	3,862.11	21.54	32.52	1.95	64.74
std	7.19	1,414.76	28.10	82.70	2.03	98.26
min	-3.10	26.28	0.00	0.00	1.00	1.00
max	7.28	3,055.05	4.20	1.50	1.00	22.98
Q1	13.34	3,766.80	11.00	9.00	1.00	37.10
Q2	18.02	4,857.53	26.00	30.20	2.00	69.70
Q3	29.30	8,760.00	171.68	1,300.00	12.00	1,804.40

A correlation analysis was performed by building a Pearson’s correlation matrix (Figure S2 from Extended data ^15^). It was observed that there was a moderate correlation (0.4) between TOW and temperature, as well as a similar moderate correlation (0.45) between time and corrosion.

### 3.2 Evaluation of the developed models

**3.2.1 Model architectures**

The models were developed sequentially in increasing complexity. The metrics for comparing the different models among them were R ^2^ and RMSE. The first model that was built was the MLR. The regression coefficients were b0 = -0.0809175; b1 = 0.0945785; b2 = 0.153686; b3 = 0.287786; b4 = 0.56445; b5 = 0.276023. After building the model, an inverse normalization was applied for obtaining absolute errors. The accuracy of the MLR based on the regression coefficients obtained is described in Table 7.

**Table 7.** Summary of model architectures development.

Model	R ^2^ training	R ^2^ validation	RMSE training	RMSE validation	Data normalized	Number of hyperparameter evaluated
**MLR**	0.50	0.47	51	58	yes	0
**PR**	0.65	0.61	43	46	yes	1
**DTR**	0.85	0.43	29	45	no	2
**RFR**	0.89	0.70	23	36	no	3
**SVR**	0.79	0.72	33	39	yes	4
**MLPR**	0.78	0.76	31	41	yes	6

For the PR algorithm, first the grade of polynomial regression that fits better the model was evaluated. Grades from 2 to 4 were considered for development of the PR model architecture. Figure S3 from Extended data ^15^ shows how increasing the polynomial degree increases the RMSE in the validation dataset while remaining relatively constant in the training dataset. This is probably due to data overfitting to the training dataset at higher polynomial grades, implying a better model fit to the training dataset but less predictive power once applied to the validation dataset, resulting in such high RMSE values. Therefore, the best predictive configuration is obtained for a polynomial grade 2. Table 7 describes the accuracy metrics obtained in the PR model with grade 2 as a fixed hyperparameter. Comparing with MLR validation (R ^2^= 0.47 and RMSE of 58), the accuracy metrics of the PR are better (R ^2^= 0.61 and RMSE 46) which is expected as PR models can capture better non-linear behaviour.

The best fit values obtained from the gridsearch algorithm for developing the DTR model are listed in Table 8. The results indicate that the optimal maximum depth of the tree is 8 and the minimum number of samples evaluated in each node are 10. For deciding which hyperparameter values must be adopted, the gridsearch algorithm was launched 100 times and the mode of the hyperparameter values was chosen.

**Table 8.** Best fit values of the hyperparameters adjusted for the decision tree regression (DTR) model.

Hyperparameter	Best fit value
**Maximum depth**	8
**Minimum number of samples**	10

The DTR model was built with the obtained optimal hyperparameters. The accuracy metrics of the model are listed in Table 7. When compared to the PR model, the DTR model fitted the training data better with a R ^2^ of 0.85 (compared with a R ^2^ of 0.65 for the PR model). However, the predictive capability of the DTR model was worse than that of the PR model, as evidenced by a R ^2^ of only 0.43 when applied to the validation data. This behaviour is expected for DTR models because these tend to overfit to the training data, with a consequent reduction in the predictive capability when applied to a different dataset (e.g., when applied to the validation dataset) ^36^.

In the case of the RFR algorithm, three hyperparameters were studied. The RFR algorithm is composed of several decision trees, therefore two of the adjusted hyperparameters were the same as for the DTR model (maximum tree depth and minimum number of samples evaluated in each node). The additional hyperparameter in comparison with a single decision tree was the number of estimators (i.e., the number of decision trees). The RFR hyperparameter configuration obtained following the methodology exposed is listed in Table 9.

**Table 9.** Best fit values of the hyperparameters adjusted for the random forest regressor (RFR) model.

Hyperparameter	Best fit value
**Maximum depth**	15
**Minimum number of samples**	6
**Number of estimators**	30

The accuracy of the model with the architecture described is shown in Table 7 where it can be observed that the predictive capabilities of the RFR model improve all the previous models. The best predictive capabilities are reflected in the accuracy metrics (R ^2^ and RMSE), where R ^2^ is the third highest value of all the models (0.70 vs 0.72 and 0.76), indicating a good fit between real and predicted values; while RMSE, as the main parameter considered for comparison of model improvement, is the lowest among all the models (RMSE = 36), indicating that the mean error average for the predicted values is the smallest. Figure S4 from Extended data ^15^ shows how well the predictive values represent the real ones. As an additional application, the RFR allows obtaining the degree of importance of each input variable to the model. In this case, it was found that for the dataset created, chloride deposition was the most relevant feature in the prediction of corrosion (Figure S5 from Extended data ^15^).

For the SVR model, the gridsearch algorithm was ran only for 5 times due to the high computational times required for each run. Once the best fit values of the hyperparameters were found ( Table 10), the metrics from the best values fitted were obtained and summarized in Table 7. In this case, the SVR model fits the training data (R ^2^ training = 0.79) worse than the RFR model (R ^2^ training = 0.89), but the predictive capabilities are better as shown by the better fit of the validation data (R ^2^ validation = 0.72 for SVR and R ^2^ validation = 0.70 for RFR). Figure S6 shows the adjustment between real and predicted values.

**Table 10.** Best fit values of the hyperparameters adjusted for the support vector regressor (SVR) model.

Hyperparameter	Best fit value
**Kernel**	rbf
**C**	6.00
**Gamma**	3.40
**Epsilon**	0.03

The last ML model tested was MLPR. In this case, as mentioned in methodology section, the computational time was so high for evaluating multiple times the best configuration applying the gridsearch algorithm. Having in mind that, the gridsearch algorithm was employed once (without iterating each time the algorithm with a new random dataset splitting as before). Once the model architecture was decided ( Table 11), the MLPR was trained and validated with the chosen hyperparameters.

**Table 11.** Best fit values of the hyperparameters adjusted for the multi-layer perceptron regressor (MLPR) model.

Hyperparameter	Best fit value
**Activation function**	relu
**Learning rate**	constant
**Alpha**	0.061
**Solver**	lbfgs
**Hidden layers**	(18,50,24)

The metrics obtained from the MLPR simulation are described in Table 7. The accuracy of the MLPR model for predicting corrosion values was the highest among the tested ML algorithms (R ^2^ validation = 0.76) but the error obtained is slightly higher than that obtained with SVR model (RMSE validation = 41 for MLPR and RMSE validation = 39 for SVR) as can be observed graphically in Figure S7 from Extended data ^15^, where a comparison between predicted and real values its shown.

***3.2.2 Analysis of models’ accuracy metrics***

Table 7 summarizes the main characteristics of each of the developed ML models, as well as the computational times that have been carried out each time any of them was evaluated. The results show that the algorithm that captures the overall trend in the data more effectively is MLPR (R ^2^ validation = 0.76), although the error produced is slightly higher than SVR and RFR ( *RMSE validation* = 41 for MLPR, *RMSE validation* = 36 for RFR and *RMSE validation* = 39 for SVR) indicating that is not the most precise model minimizing the prediction error. The ability of the RFR model to aggregate the results of multiple decision trees helped it strike a balance between fitting the training data and generalizing to the validation set resulting in the lowest prediction error when compared with the other two (SVR and MLPR). Additionally, it can be observed how the computational analysis time of each of the algorithms increased as they become more complex.

### 3.3 Comparison with existing models

The collected data was used for calibrating existing corrosion models to compare if the ML model improves the models’ behavior. Two models were tested with the full dataset built for the ML algorithm. The first one was the model of ISO 9224 ^16^ that follows the following relationship:


$$D=r_{corr}t^{h}\;(5)$$

Where *D* is the CR in µm, *r _corr_* is the corrosion rate experienced for each environmental case in the first year expressed in µm/year, *t* is the time of exposure expressed in years and *h* is the metal-environment-specific time exponent calculated according to ISO 9224.

The *h* value obtained for the full dataset was 0.523, while a specific *r _corr_* value was calculated for each dataset row. The accuracy metrics of the model were *R ^2^* = -0.13 and *RMSE* = 80. Seeing these results, it can be concluded that this model has not predictive behavior with the current dataset, and it just can be applied in specific conditions where the range of the values is more limited than in the present dataset.

The second model that was tested was the Klinesmith’s model ^37^. In this model, the following corrosion relationship is adjusted through the least-squares method ^38^:


$$y=A\cdot t^{B}\cdot {\left(\frac{TOW}{C}\right)}^{D}\cdot {\left(1+\frac{SO_{2}}{E}\right)}^{F}\cdot {\left(1+\frac{Cl^{-}}{G}\right)}^{H}\cdot e^{J\cdot (T+T_{0})}\;(6)$$

Where *y* is the CR in µm; *C*, *E*, *G* and *T _0_* are the mean of each environmental parameter associated with. *A*, *B*, *D*, *F*, *H* and *J* are obtained from the least-squares method.

C, E, G and T _0 _values were 3862.11, 21.54, 32.52 and 13.93, respectively. In the case of A, B, D, F, H and J, the values obtained employing the least-squares method were 9.81, 0.58, 0.57, 0.44, 0.51 and 0.033, respectively. With all the parameters found, the accuracy metrics of the Klinesmith’s model were *R ^2^* = 0.6 and *RMSE* = 47., which are similar to the validation accuracy metrics obtained for the PR model ( *R ^2^* = 0.61, *RMSE* = 46). The Klinesmith’s model can predict corrosion behavior, but it is a less powerful tool in comparison to the four best ML models (DTR, RFR, SVR and MLPR), which have better predictive capabilities than conventional models (see Table 7).

## 4 Conclusions

In this work, we have explored various levels of ML algorithms to address the problem of atmospheric corrosion considering environmental parameters, in order to determine which of the models offers the best predictive capabilities. Starting with simpler models and progressing to more complex ones, RFR, SVR, and MLPR exhibited the best performance, with very similar accuracy metrics. Any of these models can reliably predict corrosion scenarios for environments ranging from C1 to C4 (which comprise the majority of the data) as well as C5-CX, though the latter only represents 12% of the dataset. The limited availability of data for C5-CX scenarios made these more challenging to model. Additionally, the RFR model revealed the importance of the environmental parameters on the corrosion behavior, being Cl ^-^ deposition the most influential parameter in severe corrosion. This explains why offshore environments, where Cl ^-^ is more prevalent, experience more intense corrosion compared to onshore environments, where Cl ^-^ levels are lower.

Moreover, through an extensive literature review, we have compiled a comprehensive and curated dataset containing over 800 records of atmospheric steel corrosion under various environmental conditions, including T, TOW, SO _2_ deposition rate, Cl ^-^ deposition rate and exposure duration. This dataset will be available to researchers for future studies on corrosion.

## Data Availability

Data sets that are not publicly available are not present in any form in the manuscript. The publications from where the dataset’s data was obtained are under subscription. Journal policies restrict the publication of its data, not its use in the study. For granted access to the dataset data, subscription payment is required. The dataset built for the study in Subsection *2.1.2 Construction of the Dataset* *was* fully described on it to allow readers to identify data sets as similar to the analysis data as possible. Zenodo: Development and Comparative Analysis of Machine Learning Algorithms for Predictive Atmospheric Corrosion Modeling: Extended Data ^15^. The project contains the following underlying data: Extended data.docx Data are available under Creative Commons Attribution 4.0 International
