# Supplementary material for: Development and comparative analysis of machine learning algorithms for predictive atmospheric corrosion modeling
Source: Open Res Eur. 2025 Dec 2;5:84. Originally published 2025 Mar 27. [Version 2] doi: 10.12688/openreseurope.19770.2 (PMC12869018; doi:10.12688/openreseurope.19770.2)
Supplement: Peer review history

# Reviewer report: Reviewer response for version 2

**DOI:** 10.21956/openreseurope.23871.r65332

**Published:** 2026-04-06

**Authors:** Atwakyire Moses

**Affiliations:** 1 Kabale University, Kabale, Uganda

Approved

Is the study design appropriate and does the work have academic merit?

Yes

Is the work clearly and accurately presented and does it cite the current literature?

Partly

If applicable, is the statistical analysis and its interpretation appropriate?

Partly

Are all the source data underlying the results available to ensure full reproducibility?

Partly

Are the conclusions drawn adequately supported by the results?

Yes

Are sufficient details of methods and analysis provided to allow replication by others?

Yes

Reviewer Expertise:

Material science, Machine learning, corrosion Testing,

I confirm that I have read this submission and believe that I have an appropriate level of expertise to confirm that it is of an acceptable scientific standard.

# Reviewer report: Reviewer response for version 2

**DOI:** 10.21956/openreseurope.23871.r67945

**Published:** 2026-02-03

**Authors:** Zhongheng Fu

**Affiliations:** 1 University of Science and Technology Beijing, Beijing, Beijing, China

The authors have carefully addressed the comments raised by the previous reviewers. Prior to publication, I recommend that the authors incorporate an interpretability analysis of the machine-learning results (e.g., feature importance or attribution analysis) to improve the transparency of the predictive model and enhance the appeal of the manuscript to readers in the corrosion science community.

Is the study design appropriate and does the work have academic merit?

Yes

Is the work clearly and accurately presented and does it cite the current literature?

Yes

If applicable, is the statistical analysis and its interpretation appropriate?

Yes

Are all the source data underlying the results available to ensure full reproducibility?

Partly

Are the conclusions drawn adequately supported by the results?

Yes

Are sufficient details of methods and analysis provided to allow replication by others?

Yes

Reviewer Expertise:

Corrosion science, Machine learning

I confirm that I have read this submission and believe that I have an appropriate level of expertise to confirm that it is of an acceptable scientific standard, however I have significant reservations, as outlined above.

# Reviewer report: Reviewer response for version 2

**DOI:** 10.21956/openreseurope.23871.r65333

**Published:** 2025-12-30

**Authors:** Leonardo Bertolucci Coelho

**Affiliations:** 1 Université Libre de Bruxelles,Belgium, Bruxelles, Belgium

Approved

Is the study design appropriate and does the work have academic merit?

Yes

Is the work clearly and accurately presented and does it cite the current literature?

Partly

If applicable, is the statistical analysis and its interpretation appropriate?

Partly

Are all the source data underlying the results available to ensure full reproducibility?

Partly

Are the conclusions drawn adequately supported by the results?

Yes

Are sufficient details of methods and analysis provided to allow replication by others?

Yes

Reviewer Expertise:

corrosion, AI, machine learning

I confirm that I have read this submission and believe that I have an appropriate level of expertise to confirm that it is of an acceptable scientific standard.

# Reviewer report: Reviewer response for version 1

**DOI:** 10.21956/openreseurope.21388.r52605

**Published:** 2025-04-18

**Authors:** Leonardo Bertolucci Coelho

**Affiliations:** 1 Université Libre de Bruxelles,Belgium, Bruxelles, Belgium

Strengths:

- Comprehensive standardized dataset from diverse sources
- Systematic evaluation of multiple ML algorithms
- Clear methodology for model development and validation
- Practical focus on corrosion prediction
- Effective comparison with conventional models

Major Comments:

1. Literature Integration: Incorporate more recent peer-reviewed studies on ML for corrosion prediction, especially those related to similar conditions or methods.
2. Statistical Analysis: Add formal statistical tests (e.g., p-values or confidence intervals) to support model performance comparisons.
3. Performance at High Corrosion Rates: Directly address decreased accuracy at higher corrosion rates, discuss possible reasons, and suggest mitigation strategies relevant for offshore environments.
4. Dataset Limitations: Clearly state the limitation regarding low representation of C5-CX categories (12% of the dataset) and propose strategies for addressing this in future work.
5. Data Availability: Provide clearer details or justifications on dataset accessibility for reproducibility purposes.

Minor Comments:

- Correct typographical error "attempting methods" in Methods section.
- Expand discussion on RFR feature importance, particularly regarding chloride deposition.
- Ensure consistency in formatting tables and figures.
- Clarify dataset preprocessing steps, specifically handling of units and measurement methods.
- Include a brief discussion on computational efficiency for practical application.

Conclusion: The manuscript offers valuable insights into ML-based corrosion prediction. Addressing the suggested revisions, particularly statistical analysis, high corrosion rate performance, and literature integration, will significantly enhance its suitability for indexing.

Is the study design appropriate and does the work have academic merit?

Yes

Is the work clearly and accurately presented and does it cite the current literature?

Partly

If applicable, is the statistical analysis and its interpretation appropriate?

Partly

Are all the source data underlying the results available to ensure full reproducibility?

Partly

Are the conclusions drawn adequately supported by the results?

Yes

Are sufficient details of methods and analysis provided to allow replication by others?

Yes

Reviewer Expertise:

corrosion, AI, machine learning

I confirm that I have read this submission and believe that I have an appropriate level of expertise to confirm that it is of an acceptable scientific standard, however I have significant reservations, as outlined above.

# Response

**Published:** 2025-11-26

**Authors:** Arturo Sánchez-Ramos

**Comment 1: **We sincerely appreciate the constructive comments of the author. We have added the following references:

 • For ML corrosion techniques: Moses, A., Peng, X., Wang, S. et al. Unraveling Magnesium Alloy Corrosion Patterns Through Unsupervised Machine Learning: Exploring Clustering Techniques for Enhanced Insight. JOM 76, 4388–4403 (2024). https://doi.org/10.1007/s11837-024-06674-4.

 • For Data Augmentation and Transfer Learning: Salma Fayaz, Syed Zubair Ahmad Shah, Nusrat Mohi ud din, Naillah Gul, Assif Assad, Advancements in Data Augmentation and Transfer Learning:A Comprehensive Survey to Address Data Scarcity Challenges, Recent Advances in Computer Science

 and Communications; Volume 17, Issue 8, Year 2024, e100124225452. DOI: 10.2174/0126662558286875231215054324

**Comment 2:** We have made an ANOVA analysis for the environmental parameters and how they affect corrosion, including p-values. The results of this analysis have been included in the revised version of the manuscript in Section 3.1, page 12.

**Comment 3:** Thank you for your constructive comment. Indeed, further discussion about the limitations of the model and the decreased accuracy at higher corrosion rates is needed. Two main reasons might be responsible for this observation: The first one is the lack of samples at the higher corrosion depths, making it harder for the algorithm to train properly at those ranges and resulting in less predictive accuracy. The second one, especially among the most complex algorithms, is related to the overfitting of the training dataset, which results in an overrepresentation of trends appearing in the training samples that are not followed outside that training sample. These two limitations have been discussed in the revised manuscript in Section 3.2 (evaluation of the models developed) and in Conclusions and some alternatives proposed for tis mitigation in future works as commented in the next comment.

**Comment 4:** Following the previous comment, the lack of data samples in C5-CX categories has been discussed as a limitation found during the development of the present work. Data augmentation and transfer learning have been proposed as possible mitigation strategies to overcome this limitation for future works in Section 4 (page. 17).

**Comment 5:** We are committed to open science, but some of the data records were sourced from non-open access articles for which we do not have redistribution rights. For this reason, the dataset has not been published but replicability has been pursued as much as possible by indicating both the origin of the sources and the methodology followed for the subsequent data cleaning and preprocessing (Section 2.2).

**Minor comments:** The minor comments have been addressed following the indications of the reviewer. We sincerely appreciate his attention to detail and constructive contribution.

# Reviewer report: Reviewer response for version 1

**DOI:** 10.21956/openreseurope.21388.r52603

**Published:** 2025-04-09

**Authors:** Atwakyire Moses

**Affiliations:** 1 Kabale University, Kabale, Uganda

**Comment: 1**

 The manuscript addresses relevant literature to some extent but would benefit from deeper integration of recent advancements in the field. In particular, the authors are encouraged to incorporate findings from the study published under Moses A et al. [2024 (Ref-1)] https://doi.org/10.1007/s11837-024-06674-4, which offers valuable insights that could enhance both the depth and contextual relevance of the current work. Additionally, the reference titled *"What is Corrosion? ECS, Accessed September 6, 2023"* is a general web source that lacks peer-review and scholarly rigor. Replacing it with the aforementioned peer-reviewed publication would improve the manuscript's credibility and better align it with academic standards.

**Comment: 2**

 While the manuscript includes mean values and standard deviations for mechanical tests, the use of statistical significance testing (e.g., ANOVA or t-tests) is not explicitly mentioned. However, the trends are clear, and the interpretation is logical and backed by the data. If feasible, the inclusion of p-values or confidence intervals can strengthen the statistical robustness of the current work.

**Comment 3**

 In the extended file data provided, The plot shows a general positive correlation between predicted and actual corrosion depths, indicating that the model captures the overall trend. However, there is a noticeable dispersion of data points around the best-fit line, particularly in the mid-to-high corrosion depth range. This suggests the model is less accurate when predicting higher corrosion levels. The presence of both under- and over-estimations (scattered points far from the line) reveals that prediction errors are not uniform across the range and these needs to be resolved.

**Comment: 4**

 The conclusion is coherent and highlights both the practical implications of the findings and the contribution to the corrosion research community. However, the conclusion could be further strengthened by more explicitly addressing the limitations of the study (e.g., data scarcity) and suggesting how future work might overcome these. For example, strategies such as data augmentation or transfer learning could be briefly mentioned. Additionally, while the provision of a publicly available dataset is available it does not provide the real dataset used in the manuscript, a more detailed mention of its potential applications (e.g., benchmarking future models or enabling domain-specific analysis) would reinforce its research value.

Is the study design appropriate and does the work have academic merit?

Yes

Is the work clearly and accurately presented and does it cite the current literature?

Partly

If applicable, is the statistical analysis and its interpretation appropriate?

Partly

Are all the source data underlying the results available to ensure full reproducibility?

Partly

Are the conclusions drawn adequately supported by the results?

Yes

Are sufficient details of methods and analysis provided to allow replication by others?

Yes

Reviewer Expertise:

Material science, Machine learning, corrosion Testing,

I confirm that I have read this submission and believe that I have an appropriate level of expertise to confirm that it is of an acceptable scientific standard, however I have significant reservations, as outlined above.

## References

[1] : Unraveling Magnesium Alloy Corrosion Patterns Through Unsupervised Machine Learning: Exploring Clustering Techniques for Enhanced Insight. *JOM* .2024;76(8) : 10.1007/s11837-024-06674-4 4388-4403 10.1007/s11837-024-06674-4

# Response

**Published:** 2025-11-26

**Authors:** Arturo Sánchez-Ramos

Comment 1: We thank the reviewer for this contribution. The reference to Moses et al. (2024) has been added to the introduction, as it provides valuable insights into corrosion pattern recognition using unsupervised machine learning. At the same time, we decided to retain the citation to the Electrochemical Society’s overview [1], as it a well-established scientific journal in the field and offers a concise and authoritative definition of corrosion. Comment 2: We have made an ANOVA analysis for the environmental parameters and how they affect the corrosion, including p-values. The results of this analysis have been included in the revised version of the manuscript in Section 3.1, page 12.

 Comment 3: This is a very pertinent comment worth of further explanation on the paper. Two main reasons might be responsible for that observation: The first one is the lack of samples at the higher corrosion depths, making it harder for the algorithm to train properly at those ranges and resulting in less predictive accuracy. The second one, especially among the most complex algorithms, is related to the overfitting of the training dataset, which results in an overrepresentation of trends appearing in the training samples that are not followed outside that training sample. These two limitations have been discussed in the revised manuscript in Section 3.2 (evaluation of the models developed) and in Conclusions.

 Comment 4: Following the suggestion from the previous and the present comment, the scarcity of data has been discussed in the conclusion section as a limitation found during the study. Data augmentation and transfer learning have been discussed as a way to overcome this current limitation in future works.

 Regarding the dataset, while we are committed to open science, we regret that we cannot share the exact dataset used in this study due to the inclusion of samples from non-open access manuscripts for which we do not have redistribution rights. However, replicability has been pursued as much as possible by indicating both the origin of the sources and the methodology followed for the subsequent data cleaning and preprocessing (Section 2.2).
